# Kahweol Induces Apoptosis in Hepatocellular Carcinoma Cells by Inhibiting the Src/mTOR/STAT3 Signaling Pathway

**DOI:** 10.3390/ijms221910509

**Published:** 2021-09-29

**Authors:** Hye-Young Seo, So-Hee Lee, Ji-Ha Lee, Jae-Ho Lee, Byoung Kuk Jang, Mi Kyung Kim

**Affiliations:** 1 Department of Internal Medicine, School of Medicine, Institute for Medical Science, Keimyung University, Daegu 42601, Korea; seo568@hanmail.net (H.-Y.S.); jy16162727@naver.com (S.-H.L.); jihain10@gmail.com (J.-H.L.); 2 Department of Anatomy, Keimyung University School of Medicine, Dageu 42601, Korea; anato82@dsmc.or.kr

**Keywords:** kahweol, Src kinase, HCC, apoptosis

## Abstract

Kahweol, a coffee-specific diterpene, induces apoptosis in human cancer cells, and some targets of kahweol-mediated apoptosis have been identified. However, the specific apoptotic effects and mechanism of action of kahweol in hepatocellular carcinoma (HCC) cells are unknown. This study was performed to investigate the molecular mechanism by which kahweol induces apoptosis in HCC cells. The Src pathway is associated with apoptosis in cancer. In this study, we found that kahweol induces apoptosis by inhibiting phosphorylation of Src, and also inhibiting p-mTOR and p-STAT3. Therefore, we suggest that kahweol is a potent inhibitor of HCC cell growth.

## 1. Introduction 

Hepatocellular carcinoma (HCC) is the sixth most commonly diagnosed cancer and the third leading cause of cancer mortality worldwide [1]. Chronic hepatitis C (CHC), chronic hepatitis B (CHB), alcohol, and non-alcoholic fatty liver disease (NAFLD) are the most common causes of HCC. Despite the recent development of various antiviral agents, there are unmet needs to prevent and treat HCC [2,3,4]. A reduction in the incidence of HCC due to NAFLD is achieved through weight loss by diet control and increased physical activity. Dietary and nutritional supplements such as polyunsaturated fatty acids, vitamin D, branched-chain amino acids, and coffee can also prevent the occurrence of HCC [5].

Coffee is one of the most consumed beverages worldwide [6]. Recent meta-analysis indicates that coffee drinkers have reduced incidences of HCC and chronic liver disease [7,8]. Coffee is a mixture of many bioactive compounds. Among them, kahweol is a natural diterpene extracted from coffee beans in unfiltered coffee beverages such as espresso or boiled coffee [9]. Kahweol also has anti-inflammatory and anti-tumor progression properties [10,11].

**Figure 1:**
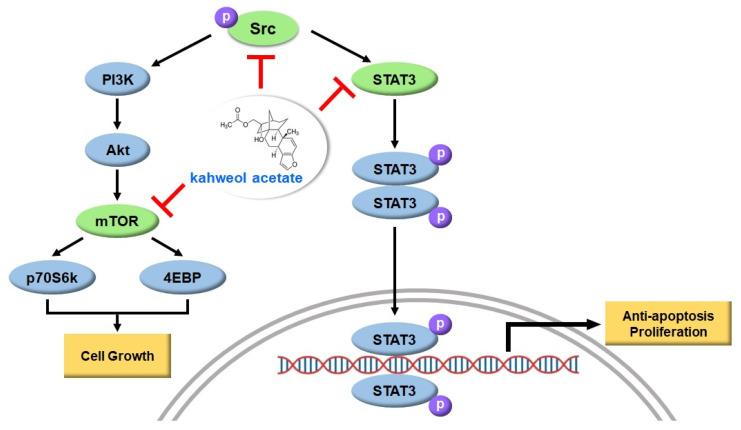
Anti-proliferative and apoptosis properties of kahweol in HCC cells. Kahweol inhibited phosphorylation of Src, mTOR, and STAT3 in HCC cells. Phosphoinositide 3-kinases (PI3K), Mammalian target of rapamycin (mTOR), 70-kDa ribosomal protein S6 kinase (p70S6K), eukaryotic translation initiation factor 4E binding protein (4EBP), Signal transducer and activator of transcription 3 (STAT3).

Src is a non-receptor tyrosine kinase originally identified as an oncogene, and it is expressed in human tumors [12,13]. Previous results have shown that increased Src expression levels may promote metastasis and invasion of colorectal cancer [14]. Additionally, Src promotes HCC growth and tumorigenesis, and inhibition of Src inhibits HCC growth [15]. In this study, we investigated the anti-tumor effects of kahweol and determined that its mechanism of action is related to the Src signaling pathway in HCC cells. Our results demonstrate that kahweol inhibits HCC cell growth by inhibiting the Src/mTOR/STAT3 signaling pathway (Figure 1).

## 2. Results

### 2.1. KahweolInhibits Cell Proliferation and Induces Apoptosis in HCC Cells

**Figure 2:**
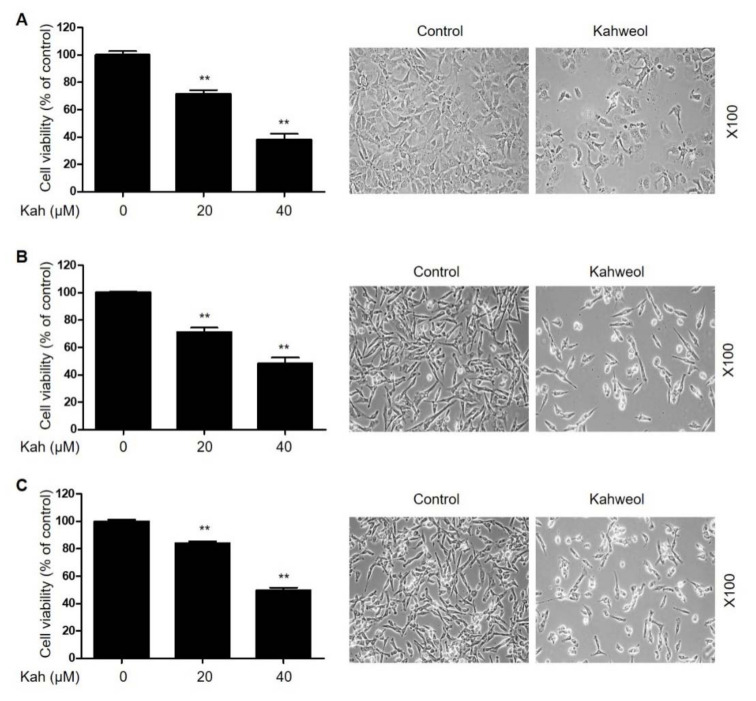
Anti-proliferative effects of kahweol in HCC cells. (**A**) Hep3B, (**B**) SNU182, and (**C**) SNU423 cells were treated with kahweol for 24 h. Cell viability was determined using a CCK-8 assay, and cell morphology was analyzed using microscopy. Cell survival is shown as a percent of control (without kahweol). ** *p* < 0.01 relative to the control (without kahweol).

**Figure 3:**
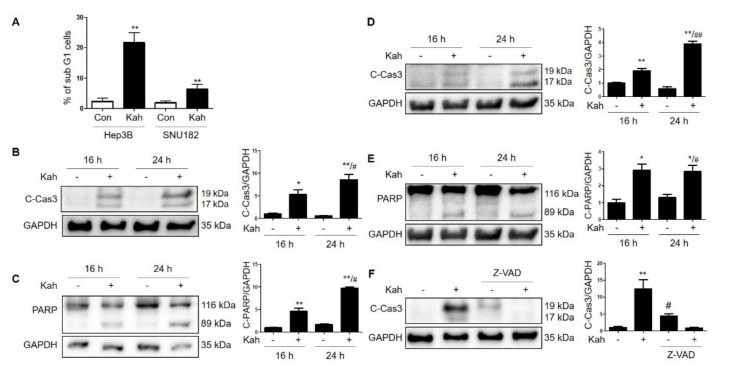
Kahweol induces apoptosis in HCC cells. (**A**) HCC cells were treated with kahweol (40 μM) for 24 h. Apoptotic cells (a sub-G1 population) were identified using flow cytometry. ** *p* < 0.01 relative to control. Western blot analyses of (**B**,**D**) cleaved caspase 3 (**B**, Hep3B cells; and **D**, SNU182 cells) and (**C**,**E**) cleaved PARP (**C**, Hep3B cells; and **E**, SNU182 cells) in HCC cells treated with kahweol. Data in the graph are represented as the mean ± SEM of three independent measurements, and graphs are normalized to 16 h control (without kahweol). * *p* < 0.05 and ** *p* < 0.01 relative to the 16 h control. ^#^ *p* < 0.05 and ^##^ *p* < 0.01 relative to the 24 h control (without kahweol). (**F**) Hep3B cells were treated with kahweol for 24 h in the presence or absence of Z-VAD-fmk (20 μM). Data in the graph are represented as the mean ± SEM of three independent measurements. ** *p* < 0.01 relative to control and ^#^ *p* < 0.05 relative to kahweol.

Because kahweol has an anti-proliferative effect in cancer cells [16], we set out to determine whether kahweol can inhibit the proliferation of HCC cells.Treatment with kahweol for 24 h significantly inhibited Hep3B, SNU182, and SNU423 proliferation in a concentration-dependent manner (Figure 2). In addition, the kahweol-treated cells exhibited a rounded morphology, contraction, and attachment loss (Figure 2). To investigate whether cell viability decreased due to the increase in apoptosis observed, the treated cells were analyzed using flow cytometry and western blotting. Kahweol markedly induced the accumulation of a sub-G1 cellular population and increased the expression levels of cleaved caspase-3 and cleaved poly [ADP-ribose] polymerase (PARP) in HCC cells (Figure 3). Activation of caspase-3 was completely prevented when we treated the cells with the pan-caspase inhibitor z-VAD-fmk (Figure 3F). These results suggest that treatment with kahweol induces caspase-dependent apoptosis in HCC cells.

### 2.2. Kahweol Attenuates Src Phosphorylation in HCC Cells

**Figure 4:**
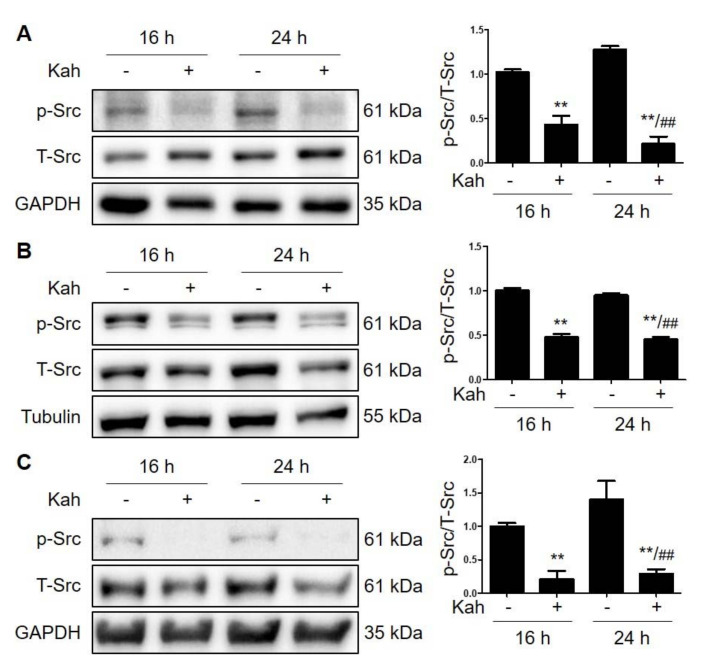
Kahweol decreases the level of p-Src expression in HCC cells. (**A**) Hep3B, (**B**) SNU182, and (**C**) SNU423 cells were treated with 40 μM kahweol for 24 h. Western blot analyses show the effects of kahweol on p-Src expression levels. Data in the graph are represented as the mean ± SEM of three independent measurements, and graphs are normalized to 16h control (without kahweol). ** *p* < 0.01 relative to the 16 h control. ^##^ *p* < 0.01 relative to the 24 h control (without kahweol).

Src kinases are involved in multiple cellular processes, including proliferation and apoptosis [17]. In addition, downregulation of phosphorylated-Src (p-Src) expression inhibits cell growth and induces apoptosis in HCC cells [18,19]. Next, we investigated whether kahweol regulates p-Src expression levels. As shown in Figure 4, p-Src was expressed in HCC cells, but kahweol decreased this expression level. Next, we treated the cells with the Src inhibitor PP2 and investigated the changes in Src levels and apoptosis. PP2 almost completely blocked p-Src expression and increased the level of cleaved-caspase3 (Figure S1). These results suggest that the apoptotic effect of kahweol is due to a decrease in p-Src expression.

### 2.3. Kahweol Attenuates mTOR Activation in HCC Cells

**Figure 5:**
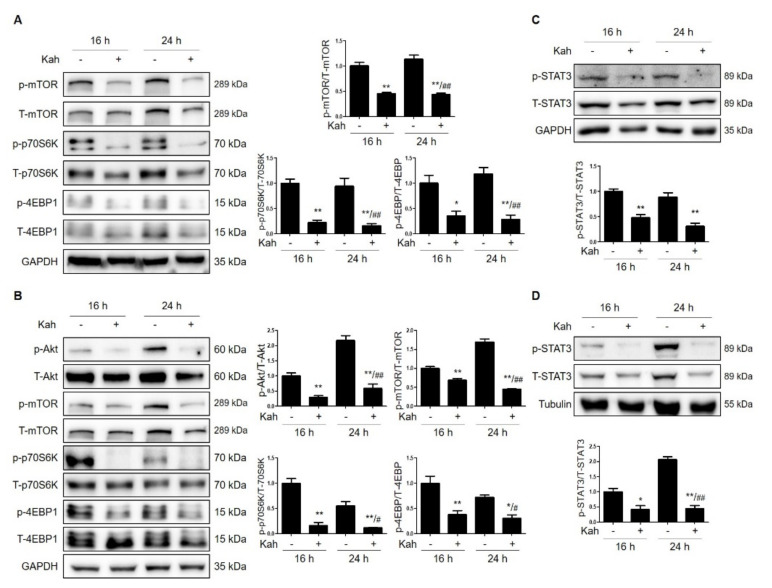
Kahweol blocks mTOR activation and decreases the level of p-STAT3 expression in HCC cells. (**A**) Hep3B and (**B**) SNU182 cells were treated with 40 μM kahweol for 24 h. Western blot analyses show the effects of kahweol on the expression levels of p-Akt, p-mTOR, p-70S6K, and p-4EBP1. Data in the graph are represented as the mean ± SEM of three independent measurements, and graphs are normalized to 16 h control (without kahweol). * *p* < 0.05 and ** *p* < 0.01 relative to the 16 h control. ^#^ *p* < 0.05 and ^##^ *p*< 0.01 relative to the 24 h control (without kahweol). (**C**) Hep3B and (**D**) SNU182 cells were treated with 40 μM kahweol for 24 h. Western blot analyses show the effects of kahweol on the expression levels of p-STAT3. Data in the graph are represented as the mean ± SEM of three independent measurements, and graphs are normalized to 16 h control (without kahweol). * *p* < 0.05 and ** *p* < 0.01 relative to the 16 h control. ^##^ *p* < 0.01 relative to the 24 h control (without kahweol).

Next, we investigated whether kahweol inhibits the Src signaling pathway. As shown in Figure 5, treatment with kahweol blocked the expression of p-Akt, p-mTOR, p-p70S6K, and p-4EBP1 in Hep3B (Figure 5A) and SNU182 (Figure 5B) cells. In addition, we confirmed that PP2 inhibited the Src signaling pathway by reducing the expression levels of pAkt, p-mTOR, p-p70S6K, and p-4EBP1 in Hep3B and SNU182 cells (Figure S2). These results suggest that kahweol suppresses p-Src expression and inhibits the Src signaling pathway.

### 2.4. Kahweol Attenuates STAT3 Phosphorylation in HCC Cells

Kahweol increasescancer cell apoptosis and blocks STAT3 phosphorylation [20]. STAT3 is one of the Src signaling pathways; therefore, we set out to determine whether kahweol-mediated inhibition of Src induces a decrease in p-STAT3. We found that kahweol treatment decreased the expression level of p-STAT3 in Hep3B (Figure 5C) and SNU182 (Figure 5D) cells. These results suggest that kahweol inhibits p-Src expression and consequently also decreases Src/STAT3 signaling.

### 2.5. Src mRNA Expressions in HCC by TCGA database

**Figure 6:**
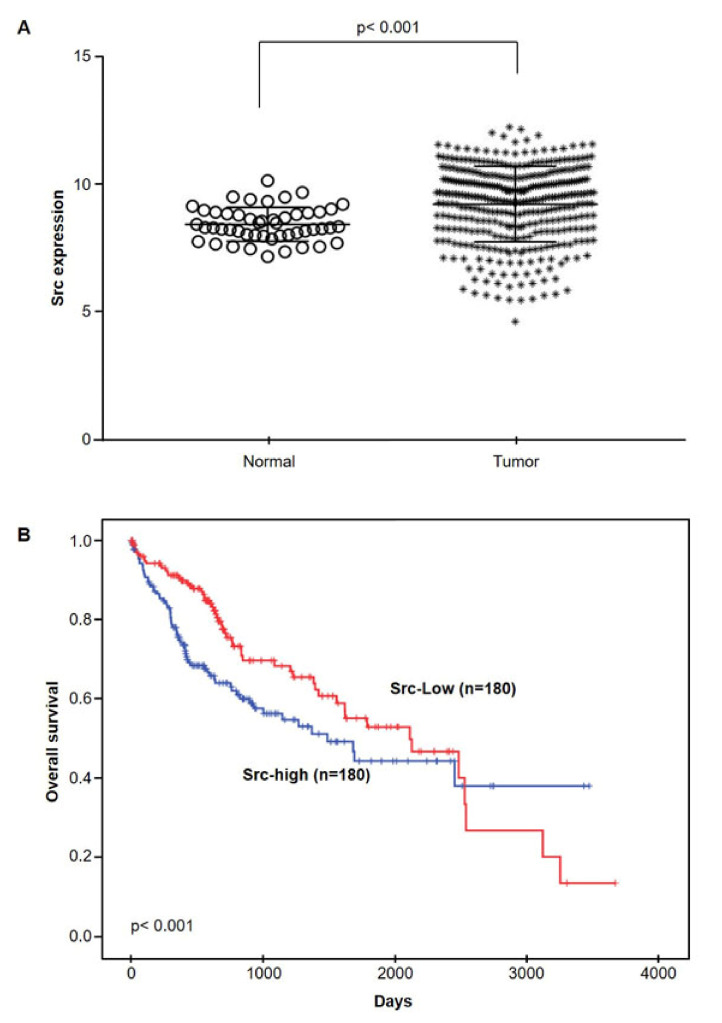
The correlation between Src overexpression and prognosis in hepatocellular carcinoma (HCC) patients. (**A**) The expression level of Src in HCC tissues (from the TCGA data analysis). (**B**) The overall survival rates for the Src high- and low-expression groups were obtained from the TCGA.

To examine the relationship between HCC cells and Src, we used the publicly available HCC dataset from the TCGA database. Src transcription in HCC cells was significantly higher than that observed in non-cancerous tissue (Figure 6A). Next, we evaluated the survival of HCC cells to determine the prognostic value of Src mRNA transcription levels. Univariate survival analysis showed that patients with higher Src transcription levels had shorter overall survival rates (1817.36 vs. 1946.76 days, *p* = 0.027) (Figure 6B). In addition, we performed quantitative correlation analysis with clinical parameters to evaluate which factors were associated with Src transcription. Src and Akt1 transcription levels were weakly negatively associated (r = −0.165 and *p* = 0.002), and STAT3 and Akt1 transcription levels were positively correlated with each other (r = 0.104 and *p* = 0.048) (Table S1 and Figure S3). Hence, the clinical data from patients with HCC also showed a close association between Src transcription levels and HCC diagnosis.

## 3. Discussion

This study evaluated the anti-cancer activity of kahweol and the pathways it affected in HCC cells. Kahweol induces apoptosis in HCC cells by inhibiting the Src/mTOR/STAT3 signaling pathway.

Kahweol inhibits tumor cell activity and proliferation, and can induce apoptosis. It has anti-tumor effects in breast cancer cells, produces cytotoxicity effects in colorectal cancer cells, and has apoptotic and anti-proliferative effects in renal cells [16,21,22,23]. Kahweol produces an apoptotic effect in cancer cells by regulating cyclins, the apoptosis-related proteins Sp1 and Akt, and the ERK/JNK pathway [24,25,26]. However, few studies have investigated apoptosis and the mechanism of action of kahweol in HCC. In this study, we show that kahweol decreases cell proliferation and induces apoptosis in HCC cells.

Src is a therapeutic target involved in tumor growth and metastasis that mediates intracellular signaling [27]. The expression of Src is elevated in HCC, and this increase is associated with HCC pathogenesis [28]. In addition, increased expression levels of Src are associated with a poor prognosis for patients with HCC. The inhibition of Src expression in HCC cells inhibits cell proliferation, promotes apoptosis, and induces potent anti-tumor effects [19]. We analyzed data from the TCGA and found that the expression levels of Src were higher in patients with HCC, and patients with higher Src expression levels had shorter overall survival (Figure 6), which is in agreement with these results. Our previous study showed that kahweol treatment decreased phosphorylation of Src. Therefore, to elucidate the mechanism underlying the anti-tumor activity of kahweol in HCC cells, we investigated whether kahweol downregulates Src expression. Herein, we show that kahweol significantly inhibits the expression of p-Src in HCC cells. Src participates in signaling pathways essential for cell proliferation and survival by phosphorylating proteins in the mammalian target of rapamycin (mTOR) and STAT3 pathways [12]. Activated mTOR phosphorylates 4E-binding protein (4E-BP1) and p70S6K translation initiation factor, increasing the synthesis of numerous proteins required for cell angiogenesis, cell proliferation and growth [19,29]. mTOR is also upregulated in HCC cells, but mTOR inhibitors alone induce limited inhibition of HCC cell growth [30]. However, combining treatment using an mTOR inhibitor with an Src inhibitor decreases HCC growth [31]. In this study, we found that kahweol suppresses p-Src and p-mTOR expression, suggesting that kahweol could be used as a therapeutic agent for HCC treatment.

Phosphorylated STAT3 produces anti-apoptotic, anti-proliferation, and anti-metastatic effects in tumor cells, and a STAT3 inhibitor exhibits anti-cancer activity through inhibition of STAT3 phosphorylation [32,33]. Activation of STAT3 is detected in HCC cells, and blocking activation of STAT3 also inhibits the growth of HCC cells [34,35]. We found that kahweol also inhibits p-STAT3 expression in HCC cells, likely due to a decrease in the level of p-Src, which is upstream of STAT3.

In conclusion, kahweol induces apoptosis in HCC cells by inhibiting Src, which is involved in the mTOR and STAT3 pathways. Hence, our findings suggest that kahweol could be an effective therapeutic agent for the treatment of HCC (Figure 1).

## 4. Materials and Methods

### 4.1. The Cancer Genome Atlas (TCGA) Data Analysis

We investigated the publically available TCGA datasets, and its data were downloaded from the TCGA Data Portal at https://tcga-data.nci.nih.gov/tcga/ (accessed on 14 July 2021). The microarray and RNA-Seq experiments and clinical data were downloaded directly from the TCGA website.

### 4.2. Chemicals

Kahweol acetate was purchased from LKT laboratories Inc. (St. Paul, MN, USA, 81760-47-6). A 40 mM solution of kahweol was initially prepared in DMSO, stored as small aliquots at −20 °C, and thawed and diluted in cell culture medium, as required. The anti-cleaved-caspase 3 (9664S,1:2000), anti-PARP (9542S,1:2000), anti-phospho-Src(2101S,Y416), anti-phospho-mTOR (S2448) (2971S1:1000), anti-phospho-p70S6K(T389) (9206S,1:1000), anti-phospho-4EBP1(T37/46) (2855S,1:5000), anti-phospho-Akt (S473) (9271S,1:2000), anti-phospho-STAT3(Y705) (9138S,1:4000), beta-tubulin (2146S,1:5000), anti-GAPDH (2118S,1:5000), anti-rabbit (7074P2,1:5000~1:10,000), and anti-mouse (7076P2,1:5000~1:10,000) were purchased from Cell Signaling Technology (Beverly, MA, USA).

### 4.3. Cell Culture

The human HCC cell lines Hep3B, SNU182, and SNU423 were purchased from the American Type Culture Collection (Manassas, VA, USA), and these cells were cultured at 37 °C with 5% CO_2_. Hep3B was cultured in MEM media, and SNU182 and SNU423 were cultured in RPMI1640. The media were supplemented with a 1% penicillin/streptomycin solution and 10% fetal bovine serum (FBS, Hyclone, Logan, UT, USA). Cells were treated with chemicals in 0.5% FBS with (kah+) or without kahweol (kah−), and proteins were isolated as described below.

### 4.4. Cell Viability Assay

Cell viability was analyzed using a Cell Counting Kit-8 (CCK-8, Dojindo, Kumamoto, Japan) assay. Cells were seeded 5 × 10^3^/well into a 96-well plate in 100 μL of media for the cells to attach. They were then left untreated (control) or treated with kahweol for 24 h. Subsequently, 10 μL of a CCK-8 solution was added to each well, and the cells were incubated for an additional 2 h. The absorbance in each well was measured at 450 nm on a spectrophotometer.

### 4.5. Flow Cytometry Analysis

Cells were suspended in 100 μL of PBS, and ethanol (200 μL) was added while vortexing. Subsequently, the cells were incubated at 4 °C for 1 h, washed with PBS, resuspended in 250 μL of a 1.12% sodium citrate buffer containing 12.5 μg RNase, and were then incubated at 37 °C for a further 30 min. The cellular DNA was stained by incubating the cells with 250 μL of propidium iodide (50 μL/mL) for 30 min. The stained cells were analyzed using fluorescence-activated cell sorting on a FACS can flow cytometer, and the relative DNA content was determined based on the red fluorescence intensity observed.

### 4.6. Western Blot Analysis

After being washed with PBS, cells were harvested and incubated with the RIPA buffer (Thermo Scientific, Waltham, MA, USA) containing protease/phosphatase inhibitors (Inhibitor Cocktail solution; genDEPOT, Katy, TX, USA). The cells were lysed on ice for 30 min, and lysate was collected by centrifugation at 13,000 rpm for 10 min. Protein concentrations were determined using a BCA assay (Thermo Scientific, Waltham, MA, USA). Equal amounts of solubilized proteins were separated using SDS-PAGE, and were subsequently transferred to PVDF membranes (Mil-lipore, Billerica, MA, USA). The membranes were blocked with 5% skim milk prepared in TBST (Tris-buffered saline containing 0.1% Tween 20). After washing (TBST), the membrane was incubated with primary antibodies. After washing again, the membrane was incubated with HRP-conjugated secondary antibody. Signals were visualized using the Clarity Western ECL substrate kit (Bio-Rad, Richmond, CA, USA). The membranes were re-probed with an anti-GAPDH or anti-tubulin antibody to verify that an equal amount of protein was loaded in each lane. Signal intensities were quantified using densitometry with the ImageJ software (version 1.52a) (NIH, Bethesda, MD, USA).

### 4.7. Statistical Analysis

A chi-square test and the Mann–Whitney U test were used to analyze the association between variables. Univariate survival analysis was constructed using the log-rank test with a Kaplan–Meier curve. Overall survival was defined as the time between diagnosis and mortality. The correlations between mRNA transcription levels and clinicopathologic parameters were assessed using Pearson’s correlation coefficient analysis. Experimental statistical analyses were performed with one-way ANOVAs with the Bonferroni correction. A *p*-value of < 0.05 and < 0.01 was considered statistically significant. Data are presented as the mean ± SEM. All experiments were performed at least three times.

## Data Availability

http://www.oncolnc.org/ (accessed on 6 September 2021).

## Supplementary Materials

The following are available online at https://www.mdpi.com/article/10.3390/ijms221910509/s1, Table S1: Correlation between TZAP expression and the clinical parameters in CRC, Figure S1: PP2 induced apoptosis in HCC cells, Figure S2: PP2 blocks mTOR activation in HCC cells, Figure S3: The correlation among the expressions of Src and Akt in HCC tissue.
