# Supplementary material for: Kahweol Induces Apoptosis in Hepatocellular Carcinoma Cells by Inhibiting the Src/mTOR/STAT3 Signaling Pathway
Source: Int J Mol Sci. 2021 Sep 29;22(19):10509. doi: 10.3390/ijms221910509 (PMC8508869; doi:10.3390/ijms221910509)
Supplement: Supplementary file 1 [file ijms-22-10509-s001.zip › ijms-1391836-supplementary.pdf]

Table S1. Correlation between TZAP expression and the clinical parameters in CRC

		Src	STAT3	Akt1	Age	AFP
Src	R	1	-.100	-.165**	-.099	.025
	P		.058	.002	.060	.682
STAT3	R	-.100	1	.104*	.006	-.094
	P	.058		.048	.903	.119
Akt1	R	-.165**	.104*	1	.116*	-.025
	P	.002	.048		.027	.677
Age	R	-.099	.006	.116*	1	.075
	P	.060	.903	.027		.212

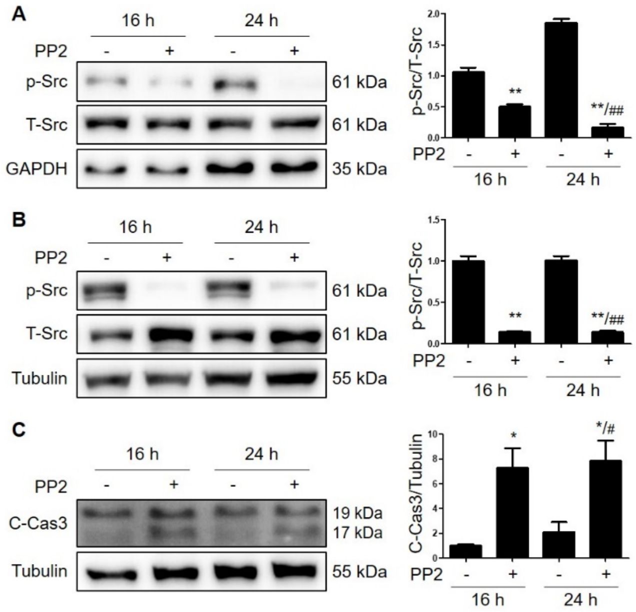

Figure S1. PP2 induced apoptosis in HCC cells. (A) Hep3B, (B) SNU182, and (C) SNU423 cells were treated with 10 μM PP2 for 24 h. Western blot analyses show the effects of PP2 on cleaved caspase 3 expression levels. *P<0.05 and **P<0.01 relative to the 16 h control. #P<0.05 and ##P<0.01 relative to the 24 h control (without kahweol).

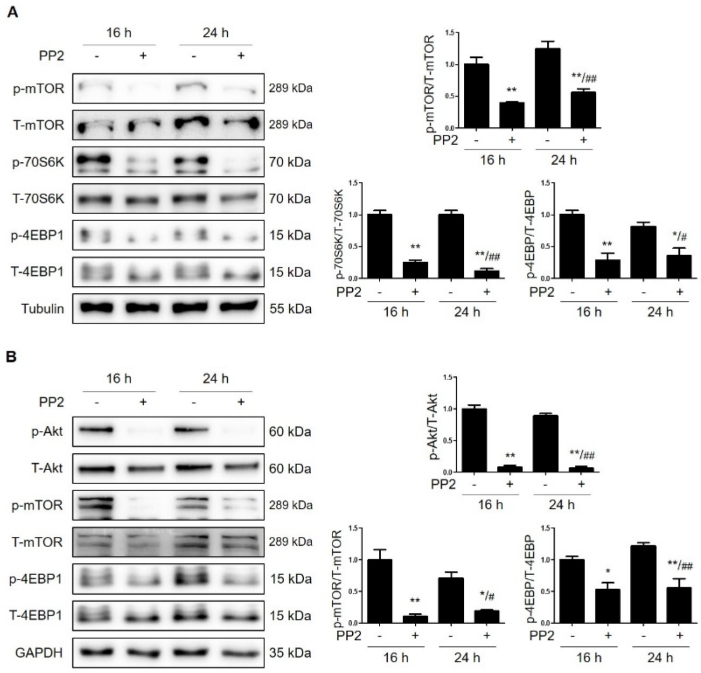

Figure S2. PP2 blocks mTOR activation in HCC cells. (A) Hep3B and (B) SNU182 cells were treated with 10 μM PP2 for 24 h. Western blot analyses showing the effects of PP2 on p-Akt, p-mTOR, p-p70S6K, and p-4EBP1 expression levels. *P<0.05 and **P<0.01 relative to the 16 h control. #P<0.05 and ##P<0.01 relative to the 24 h control (without kahweol).

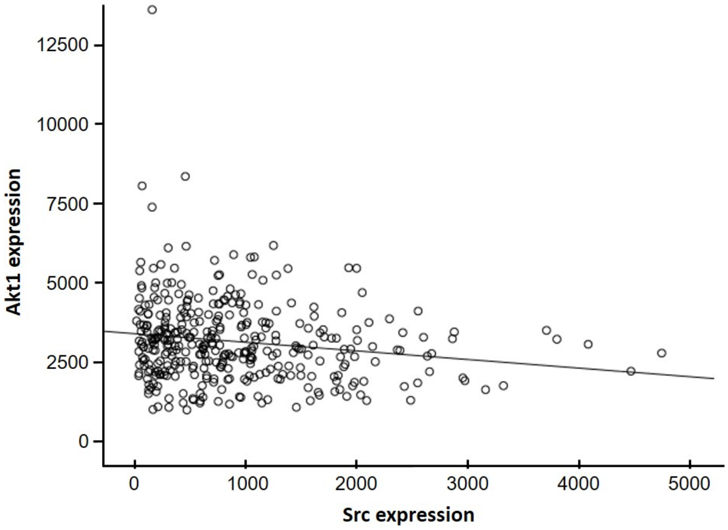

Figure S3. The correlation among the expressions of Src and Akt in HCC tissue. TCGA data showed that Src had a positive correlation with Akt.
